# The pathology of X-linked adrenoleukodystrophy: tissue specific changes as a clue to pathophysiology

**DOI:** 10.1186/s13023-024-03105-0

**Published:** 2024-03-28

**Authors:** Hemmo A.F. Yska, Marc Engelen, Marianna Bugiani

**Affiliations:** 1 Department of Child Neurology, Amsterdam Leukodystrophy Center, Emma Children’s Hospital, Amsterdam UMC location University of Amsterdam, Amsterdam Neuroscience, Amsterdam, The Netherlands; 2 Department of Pediatrics/Child Neurology, VU University Medical Centre, Amsterdam Neuroscience, Amsterdam, The Netherlands; 3 Department of Pathology, VU University Medical Centre, Amsterdam Neuroscience, Amsterdam, The Netherlands

**Keywords:** Adrenoleukodystrophy, Adrenomyeloneuropathy, Cerebral ALD, Pathology, Immunohistochemistry, ABCD1

## Abstract

Although the pathology of X-linked adrenoleukodystrophy (ALD) is well described, it represents the end-stage of neurodegeneration. It is still unclear what cell types are initially involved and what their role is in the disease process. Revisiting the seminal post-mortem studies from the 1970s can generate new hypotheses on pathophysiology. This review describes (histo)pathological changes of the brain and spinal cord in ALD. It aims at integrating older works with current insights and at providing an overarching theory on the pathophysiology of ALD. The data point to an important role for axons and glia in the pathology of both the myelopathy and leukodystrophy of ALD. In-depth pathological analyses with new techniques could help further unravel the sequence of events behind the pathology of ALD.

**Supplementary Information:**

The online version contains supplementary material available at 10.1186/s13023-024-03105-0.

## Background

X-linked adrenoleukodystrophy (ALD) is a peroxisomal disorder that affects the catabolism of very long-chain fatty acids (VLCFA) [1, 2]. ALD shows highly specific tissue vulnerability, although the biochemical defect is present in all cells of the body. The reason for this is still not well understood. Affected tissues are primarily the white matter of the central nervous system (CNS), the peripheral nervous system (PNS), the adrenal cortex and the testis. Early and prominent temporal balding is also observed in male patients, making it likely that hair follicles are also vulnerable [3].

Impaired peroxisomal beta-oxidation of VLCFA (≥C22:0) as the biochemical defect underlying ALD with VLCFA accumulation in all tissues was described many decades ago [4, 5]. In 1993, pathogenic variants in *ABCD1*, encoding the peroxisomal VLCFA-CoA transporter were identified as the cause of this defect [6]. The pathophysiology, i.e. how VLCFA accumulation causes tissue damage, is however still poorly understood. As VLCFA accumulation is the biochemical hallmark, it stands to reason that this plays a crucial role in the disease process. Indeed, observations in experimental models point to detrimental effects of VLCFA excess [7–9].

Degeneration of the long tracts in the spinal cord (the pyramidal tracts and dorsal columns) and of the zona fasciculata and reticularis of the adrenal cortex is the core pathology that is reflected in the main clinical syndromes. Additionally, a progressive leukodystrophy develops in a subset of patients. Although the histopathology in patients is well described, it is based on post-mortem studies at end-stage disease. This means that it is unclear what cell types are initially involved and are most central to the disease process. There has been much focus on the pathology of ALD in the 1970s [10–14]. Later, researchers have focused more on new model systems as these became available [15–18]. Revisiting the early pivotal pathological studies on patients can help understanding ALD and generate new hypotheses. With the advent of induced pluripotent derived neural cell models and brain organoids, there are now new opportunities for further studies.

We searched the literature for studies describing (histo)pathological changes of the nervous system in ALD from the first systematic study in 1974 to the present. We included key-publications and compared older works to current insights. This review describes the molecular and cellular basis of ALD, followed by a description of pathological studies in the CNS. We aim at providing an overarching theory on the pathology of ALD, and at identifying knowledge gaps to stimulate future research.

### ABCD1 and Molecular Pathology

ALD is caused by pathogenic variants in *ABCD1*, which is located on the long arm of the X chromosome (Xq28). Its gene product is expressed in a variety of tissues [3]. Currently, over 900 variants are known to be pathogenic (www.adrenoleukodystrophy.info). *ABCD1* encodes an ATP binding cassette (ABC) half-transporter (ABCD1) that is integral in the peroxisomal membrane [3, 19]. ABC-transporters dimerize forming both homodimers and heterodimers with other *ABCD*-gene products [20]. These include *ABCD2* (adrenoleukodystrophy-related protein [ALDRP]) and *ABCD3* (70 kDa peroxisomal membrane protein [PMP70]). PMP70 can partly compensate for ABCD1 loss-of-function [21]. Although ALDRP resembles ABCD1 structurally most closely and can compensate for the lack of ABCD1 by overexpression in vivo and in vitro [22–24], it has a different tissue distribution and has not been found to influence ALD disease phenotype [25, 26].

ABCD1 dysfunction leads to increased VLCFA levels by impairing very long-chain acyl-CoA transport into the peroxisome [27, 28]. There is a number of theories on how VLCFA build-up results in cell and tissue damage, although conclusive proof has never been presented. Firstly, cytosolic VLCFA are stored in complex lipids such as lysophosphatidylcholine (LPC), which disturb physiological membrane functions in mitochondria *in vitro.* This may lead to the formation of radical oxygen species (ROS) and concurrent tissue damage [29]. Secondly, VLCFA decrease phospholipid bilayer stability in high concentrations in vitro [7, 9]. In the case of myelin, dysfunction leads to a redistribution of Na+/K + transporters along axons. This increases axonal ATP consumption, eventually leading to a state of ‘virtual hypoxia’ [30], which also results in ROS formation [7, 29, 31, 32]. The interpretation of whether the presence of ROS in ALD is a primary step in its pathology is complicated by the fact that ROS are overproduced in many neurodegenerative diseases [33]. It is therefore unclear whether ROS overproduction is a cause or one of the consequences of the pathological process. Thirdly, VLCFA may exert direct cytotoxic effects. In experimental models, VLCFA administration causes oligodendrocyte and astrocyte cell death within 24 h [8]. Apart from these theories, it has been postulated that VLCFA accumulation alone may not be the only causative agent of ALD. The exact role of cholesterol metabolism, for example, is still under discussion [7].

ABCD1 is expressed in many different tissues, notably in the adrenal glands [3, 34, 35]. mRNA expression array blot analysis revealed that *ABCD1* is expressed in a large number of tissues. Our knowledge on the physical ABCD1-distribution in the CNS is mostly based on studies by Fouquet et al. and Höftberger et al. [36, 37]. Expression in the human CNS is high in fetal brain and lower in adults. In adult neurons, the highest expression of ABCD1 has been found in the pituitary gland, hypothalamus, basal nucleus of Meynert, periaqueductal grey matter and in the area of the locus coeruleus [37]. In one thoracic dorsal root ganglia sample, 40% of neurons showed positivity for ABCD1. Astrocytes and microglia are the main cell-types in the brain that stain positive for ABCD1. ABCD1 is clearly expressed in astrocyte cell processes. This is especially the case in the subcortical and cerebellar white matter, but much less so in areas typically associated to ALD white matter lesions, including the internal capsule, corpus callosum, lemniscus medialis and corticospinal tracts [36, 37]. Perivascular macrophages and (cultured) microglia also express ABCD1 in both human and mouse brain. A small proportion of oligodendrocytes in the subcortical white matter and cerebellum stains positive for ABCD1. Staining is however much less intense than for astrocytes, microglia and endothelial cells. Adult human-derived oligodendrocytes from the corpus callosum, the internal capsule and the anterior commissure that were cultured and stimulated with growth factors stained more strongly for ABCD1 than oligodendrocytes derived from other brain regions [36]. This indicates that region-specific cell heterogeneity may also influence ABCD1 expression [38]. The following sections describe the regional and cellular pathology of the myeloneuropathy and leukodystrophy of ALD.

### The myeloneuropathy of ALD

The myeloneuropathy of ALD, or “adrenomyeloneuropathy” (AMN), has been less systematically described in the literature than the leukodystrophy of ALD (“cerebral ALD”). Powers et al. reviewed the myeloneuropathy and included analyses of spinal cord samples from 5 additional patients with AMN [3]. AMN is characterized by a dying-back axonopathy of the long tracts with subsequent atrophy [39, 40]. Axonal damage initiates in the spinal cord and results in Wallerian degeneration. The axons of the neurons that run through the brain stem and cerebrum degenerate, which can lead to signal abnormalities on imaging in these regions [40]. Degeneration of the spinal cord is especially prominent in the cervical fasciculus gracilis (CFG) and the lumbar corticospinal tracts. Proprioceptive information is mainly communicated to the brain via the CFG and loss of propriocepsis is one of the core signs of the myelopathy of ALD. Myelin and axons show concurrent degenerative changes within spinal cord lesions, although axonal degeneration appears to precede myelin loss and may in some cases be more prominent. Lymphocytes are scarce and mainly perivascular; macrophages and microglia are abundantly present in the perivascular spaces. Reactive astrogliosis is sometimes present [3].

Patients with AMN without a leukodystrophy can also develop supraspinal lesions unrelated to long-tract axonopathy, such as in the cerebellar peduncles [41]. These lesions differ amongst patients, but are usually characterized by “dysmyelinative foci” and “myelin pallor”; terms no longer in use. Axons and oligodendrocytes are generally spared. Striated PAS-positive macrophages and activated microglia can be observed, but activated astrocytes and lymphocytes are absent or sparse [3, 40].

Peripheral nerves can also be affected in adult patients with ALD. Ultrastructural examination of four nerve biopsies revealed cytoplasmic inclusions in Schwann cells in two samples [42]. The other two biopsies were normal. Other observations in peripheral nerves include loss of myelinated and unmyelinated fibers and axonal fragmentation, swelling and paranodal retraction [35]. Based on neurophysiologic studies, the neuropathy in ALD appears to mainly affect small nerve fibers and is primarily axonal [43, 44].

In vitro studies by Gong and colleagues on an AMN mouse model found no signs that low doses of LPC C26:0 were intrinsically toxic to spinal cord neurons. Treatment did however induce the expression of the neuronal stress marker phosphatidylserine and axonal degeneration was observed in the presence of *ABCD1*^−/−^ microglia [45]. Microglia within spinal cord lesions were present in an activated state and treatment with LPC C26:0 resulted in clear upregulation of phagocytic markers. The authors proposed that the combined increased expression of neuronal stress markers and microglial activation could result in microglia-mediated axonal destruction by phagocytosis (Table 1).

**Table 1:** Characteristics of spinal cord lesions in adrenoleukodystrophy

Axons and myelin	Axonal degeneration equal to or greater than myelin loss.
Oligodendrocytes	Reduced in number.
Astrocytes	Isomorphic astrogliosis without reactive astrocytes.
Macrophages	PAS-positive macrophages abundant in perivascular spaces.
Microglia	Numerous activated microglia.
T-lymphocytes	Sparsely present as perivascular infiltrates.

Summary of the results presented by Powers et al. (2000). The following commercial antibodies were used for the stainings: neurofilament (NF), proteolipid protein (PLP), amyloid precursor protein (APP), glial fibrillary acidic protein (GFAP) and the microglia-macrophage markers HAM-56, CD68, 25FD, Ricinus, Lysozyme and non-specific esterase

### The leukodystrophy of ALD (Cerebral ALD)

Histopathological studies on the leukodystrophy of ALD have been performed since the late 1890’s [46]. From the 1970’s onwards, Schaumburg and colleagues provided detailed descriptions of brain autopsies, where they discriminated between 3 concentric histopathological zones of demyelination in leukodystrophy lesions [11]. From outside to inside, a “prelesional” zone contained preserved axons with apparent destruction of myelin and scattered activated phagocytes and astrocytes. The second, actively demyelinating zone contained many lipid-laden macrophages, numerous demyelinated axons, reactive astrocytes and striking perivascular lymphocytic infiltrations. Using electron microscopy, macrophages in this zone were found to contain paired electron-dense leaflets connected to lipid droplets [47]. The third and most central zone was characterized by severe gliosis, virtually complete loss of myelin and axons without any evidence of an active process. Oligodendrocytes and lymphocytes were generally absent from this region. Although the cortex is generally considered unaffected in the available literature, neuronal loss in the deeper cortical layers was described in a few severely affected patients [11]. It is important to note that modern immunohistochemical techniques were not yet available at the time of Schaumburg’s publication. Although cells morphologically resembled macrophages, they may have been microglia or migrated peripheral monocytes.

In the following decades, development of more modern techniques such as immunohistochemistry has allowed for more specific descriptions and a better understanding of ALD leukodystrophy lesions [48, 49]. An additional fourth zone of ‘normal appearing white matter’ (NAWM) was identified surrounding the prelesional zone. This zone contained a striking increase in microglia [50]. The four zones described in these pathological studies have also been correlated to quantitative magnetic resonance (MR) techniques such as spectroscopy and diffusion tensor imaging [51]. Recently, axonal damage was found to precede myelin breakdown in the prelesional zone [52].. This is in line with the finding that Neurofilament Light (NfL), a biomarker for axonal damage, is an early marker for the development of cerebral ALD [53]. Axonal degeneration therefore appears to be an early step in the sequence of pathological events in leukodystrophy lesions (Table 2).

**Table 2:** Characteristics of cerebral adrenoleukodystrophy lesions

	Normal-appearing white matter	Prelesional zone	Actively demyelinating zone	Gliotic core
Myelin	Normal.	No relevant myelin loss, myelin vacuolization.	Significant myelin breakdown. Few residual myelinated axons.	(Almost entirely) absent. Remnants vacuolized.
Axons	Normal.	Normal morphology, but immunohistochemical signs of damage^1^.	Substantial loss of axons.	(Almost entirely) absent.
Astrocytes	Normal.	Occasional reactive cells.	Numerous reactive cells, pattern of anisomorphic gliosis.	Scattered reactive astrocytes, pattern of isomorphic gliosis.
Oligodendrocytes	Normal.	Mild oligodendrocyte loss, cells have condensed nuclei.	Significant oligodendrocyte loss.	(Almost) entirely absent, altered morphology with few or no processes.
Microglia	Higher numbers with initial reactive morphology. Expression of homeostatic markers.	Almost entirely absent.	Significant microgliosis.	Return to near-normal activation state, with expression of homeostatic markers.
Macrophages	Absent.	Sparse macrophages, mainly in perivascular spaces.	Drastic increase in lipid-laden foamy macrophages containing degraded myelin fragments.	Persisting slight increase in numbers, as compared to NAWM.
Lymphocytes	Absent.	Absent.	Prominent perivascular cuffings of T-lymphocytes. Sparse B-lymphocytes and plasma cells.	Rare T-lymphocytes.

^1^ Axonal damage was assessed by staining for amyloid precursor protein (APP) and Bielschowsky silver impregnation. The presented results were extracted from studies by Schaumburg et al. (1974 and 1975), Powers et al. (2000), Ito et al. (2001), Eichler et al. (2008) and Bergner et al. (2019 and 2021)

### The inflammatory process

Demyelination is the hallmark of cerebral ALD and immune dysregulation is seen as an important element in its pathology. Inflammatory cells, specifically T-lymphocytes, are rarely observed in the advancing prelesional edge, but accrue in perivascular spaces in the actively demyelinating zone [11]. Due to this specific involvement of the immune system, Powers and colleagues proposed an inflammatory cytokine-mediated mechanism as opposed to a specific immune-mediated attack, as in multiple sclerosis (MS), as the cause for myelin destruction [48]. Brain sections of ALD patients were characterized as to the nature of inflammatory cells. Macrophages were found to be the predominant inflammatory cell-type within lesions. Reactive astrocytes and T-lymphocytes were moderately present throughout, and B-lymphocytes were generally absent. MHC class I was upregulated and mainly found on the surface of lymphocytes, microglia-like cells, oligodendrocytes and microvascular, suspected to be endothelial, cells at the active lesion edge. Reactive astrocytes stained strongly for TNF-alpha and, to a lesser extent, for IL-1 [48]. The prominent involvement of macrophages suggests an important role for the innate immune system. It remains unclear whether these effector cells are monocyte- or microglia-derived. The increased expression of MHC class I, the associated cytotoxic CD8 + T-cell response, and the potential cytotoxicity of inflammatory cytokines to oligodendrocytes may all negatively impact myelin integrity [54]. Destabilization of myelin followed by macrophage and astrocyte activation and the production of TNF-alpha, may also result in secondary lymphocyte activation [48].

Gortz et al. further investigated the inflammatory profile of leukodystrophy lesions by analyzing the influence of heat shock proteins (HSP) [55]. In MS, the combination of HSPs and IFN-gamma creates a pro-inflammatory environment in white matter lesions. Brain sections of cerebral ALD patients were stained for six different HSPs and IFN-gamma to investigate whether a similar mechanism could be observed. In ALD, increased HSP expression was observed in astrocytes, oligodendrocytes and macrophages in the prelesional, actively demyelinating and core gliotic zone as compared to NAWM. Many astrocytes in the prelesional zone expressed small HSPs, but not IFN-gamma. The authors therefore concluded that astrocytes show early signs of cellular stress in the prelesional zone and are probably involved in the process of demyelination. Due to the absence of IFN-gamma expression, however, the role of astrocytes is probably not directly related to the initiation of the inflammatory cascade.

Marchetti et al. suggested that patients with a tendency towards the production of anti-inflammatory cytokines could be protected from inflammatory derailment [56]. A genetic predisposition towards the production of inflammatory cytokines, as appears to be the case for TNF-alpha in symptomatic ALD patients, would induce a vicious inflammatory cycle leading to demyelination [57]. In line with this, one study found reduced anti-inflammatory capacity in peripheral macrophages of ALD patients [58]. Although the immunological profile of cerebral ALD points towards an important role for the immune system, agents aimed at limiting inflammation have mostly proven to be ineffective [7]. The reason for this remains elusive.

### Microglia in leukodystrophy lesions

Due to their remarkable distribution in leukodystrophy lesions, their expression of ABCD1 and their strong regulatory role to other cells in the CNS, microglia have been proposed as the missing link in the sequence of ALD pathological events [50, 59]. Microglia influence synaptic plasticity and neuron homeostasis [31]. Neurons exert inhibitory effects on microglia and axonal damage could, in turn, activate microglia [60]. The positive results of early hematopoietic stem cell transplantation (HSCT) point towards a role for microglia [61]. Inspired by the positive effect of this treatment in lysosomal diseases, Aubourg and colleagues were the first to perform HSCT in an 18-year old boy with ALD-related leukodystrophy in the early 1990’s. The authors observed a marked improvement of symptoms and MRI changes after treatment. They proposed a connection to microglia as the reason for this success. Donor monocyte-derived macrophages could migrate to the brain and limit inflammation, a task otherwise performed by healthy resident microglia [58, 61]. More recently, gene therapy with a lentiviral vector that targeted CD34 + cells had similar effects on cerebral ALD progression, thus strengthening this hypothesis [62].

In cerebral ALD, microglia are increased in the NAWM, they are in an activated and apoptotic state and are practically absent in the prelesional zone. In core gliotic areas, microglia seem to have returned to their normal distribution and re-express homeostatic markers that were previously lost [50]. Eichler et al. proposed a possible explanation for these observations. They observed microglial apoptosis in the prelesional zone of ALD brain lesions. The injection of LPC C24:0 in mouse cortices resulted in apoptosis near the injection site and a microglial distribution similar to white matter lesions in ALD. These results indicate that VLCFA may locally exert toxic effects on microglia [60]. The increase in microglia in NAWM as compared to healthy controls could be due to compensatory recruitment from more distal sites. When microglia fail to degrade VLCFA-enriched myelin fragments and undergo apoptosis, their contents may be released into surrounding tissue and cause an inflammatory reaction [32]. Fewer reports exist on the tissue distribution of microglia in ALD spinal cord lesions. One study reported decreased beta-oxidation in monocytes from patients with AMN compared to healthy controls [63]. Although microglia appear to play an important role in cerebral ALD, one argument that questions their role in spinal cord pathology is that HSCT does not prevent the development of myelopathy at an older age [64]. The role of microglia in ALD may therefore differ between brain and spinal cord. Regional heterogeneity and specialization of microglia in the brain and myelum could explain this difference [65].

## Other considerations

Pathological studies have provided important insights on the sequence of events in ALD, but several aspects remain unclear. For example, the mechanisms behind the spatiotemporal distribution of cerebral lesions are poorly understood. It is unclear why the corpus callosum and the corticospinal and dorsal tracts in the spinal cord are especially vulnerable in ALD, and why children under the age of 12 years are at higher risk to develop cerebral ALD [46]. The regional vulnerability may be explained by cell heterogeneity or differential expression of ABCD1. As the brain of children has a higher metabolic demand than adults, suboptimal functioning can have a large impact at these ages [66]. Pathogenic variants of *ABCD1* result in fluctuating microvascular blood flow [67, 68]. High flow heterogeneity can result in suboptimal tissue perfusion and lead to cell damage. The highest flow heterogeneity was found in the splenium of the corpus callosum, which is often the initial site of brain ALD lesions. Changes in flow were most prominent between the ages of 5–10 years, which coincides with the age at which patients have the highest risk to develop cerebral ALD [67]. Hemodynamic properties of the brain could therefore play an important role in cerebral ALD lesions. Another poorly understood aspect of ALD pathogenesis is its tissue-specificity. The adrenal glands and brain both contain high levels of cholesterol [7], and cholesterol accumulates in actively demyelinating regions of ALD brains [69, 70]. This observation strengthens the hypothesis of a link between cholesterol metabolism and ALD [71].

Another knowledge gap relates to the phenotypic differences between patients. It is unclear why the pathological process results in spinal cord degeneration only in some and cerebral ALD or adrenal insufficiency in others. A number of mechanisms have been proposed. Firstly, in unpublished work from our group, we show that C26:0 LPC levels correlate to disease severity. Mean plasma levels are higher in patients with leukodystrophy lesions and in patients with adrenal insufficiency. Moreover, patients with a severe myelopathy have higher plasma levels than patients with relatively mild spinal cord disease. Secondly, although ALD is generally considered a monogenic disease, several lines of evidence point towards a role for modifier genes. A clear genotype-phenotype correlation has never been established, and identical *ABCD1* pathogenic variants can even lead to different disease manifestations in identical twins [72, 73]. Modifier genes are probably able to partly compensate for the impaired VLCFA metabolism in ALD by offering alternative metabolic routes. Specific variants of the *CYP4F2* gene, which codes for an enzyme involved in an alternative VLCFA degradation pathway, for example, increase the risk of developing cerebral ALD [74]. The enzyme ELOVL1, which is located in the membrane of the endoplasmic reticulum, plays an important role in fatty acid elongation [75]. Knock-out of this gene in fibroblasts of ALD patients significantly reduced C26:0 VLCFA levels. *ELOVL1* has therefore been proposed as a *bona fide* candidate modifier gene [76]. Other genes, such as *SCD1*, have only recently been correlated to ALD manifestations and require further investigation [77]. Our understanding of the influence of these genes will likely increase in the coming years due to the increasing diagnostic use of next generation sequencing techniques. Thirdly, as active brain lesions show gadolinium contrast enhancement on MRI, the blood-brain barrier (BBB) conceivably plays an important role in the development of inflammatory brain lesions [78]. Numerous studies correlated traumatic head injury to the clinical onset of the leukodystrophy [79], and lesions of patients receiving HSCT often progress before arresting when patients start to clinically improve [80]. Both trauma and HSCT negatively impact the BBB. Some time after HSCT, contrast enhancement decreases, which suggests BBB reconstitution [81]. The recovery of the BBB has therefore been argued to play a role in arrested cerebral ALD. Last, a promising field of research, which requires further attention is epigenetics. A recent study on patients with cerebral ALD found increased methylation patterns of genes related to remyelination, which would inhibit remyelination potential [82].

## Conclusions

**Fig. 1:**
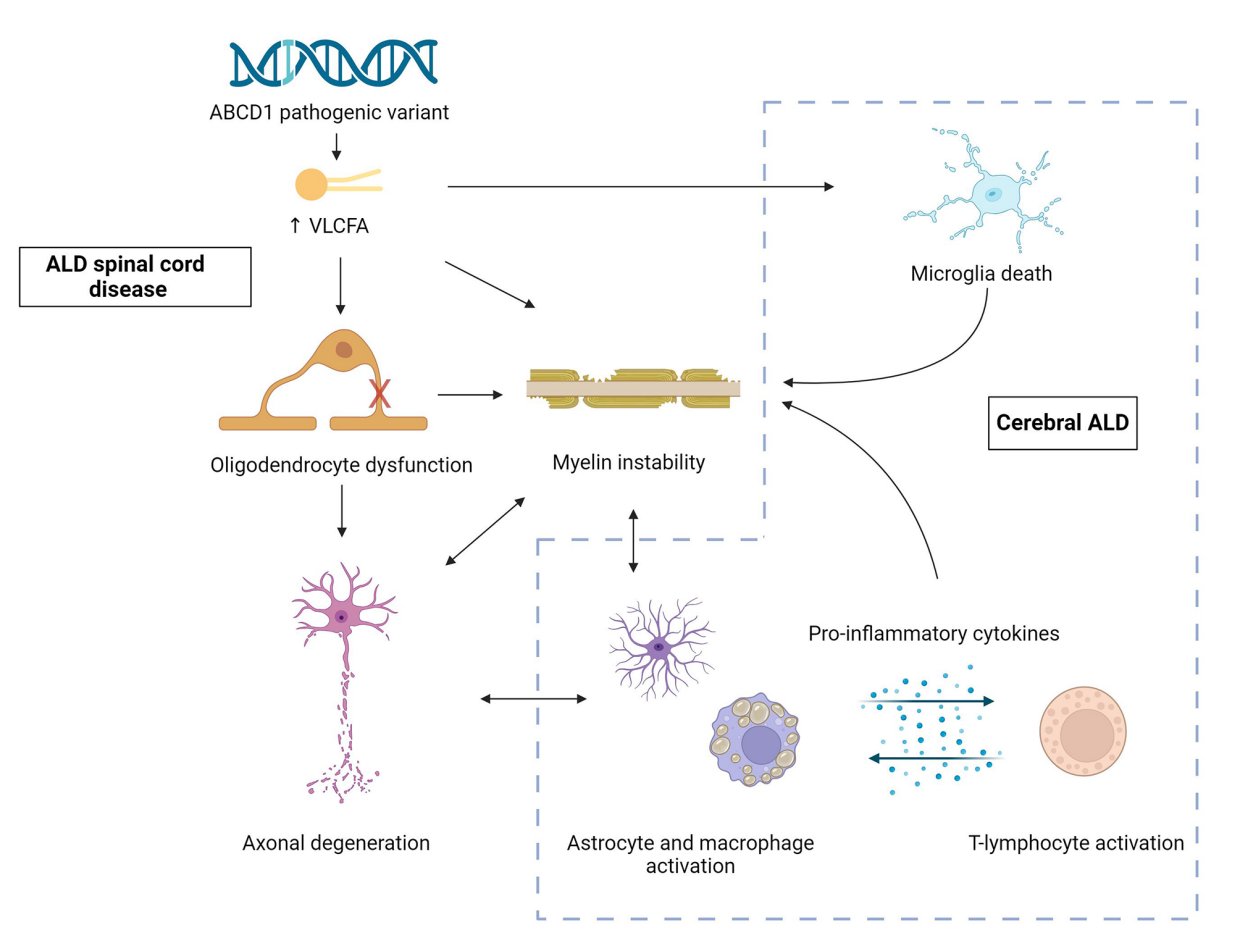
A working model for the pathology of adrenoleukodystrophy VLCFA: very long-chain fatty acids. The spinal cord disease of ALD is at the core of its pathology and is characterized by disintegration of the axon-myelin unit and oligodendrocyte dysfunction. A subset of patients develops cerebral ALD, which is characterized by astrocyte activation and peripheral macrophage recruitment. These cells produce pro-inflammatory cytokines that lead to lymphocyte activation and further damage to the axon-myelin unit. Microglia directly undergo apoptosis under the influence of very long-chain fatty acids. This figure was created with Biorender

The pathology of the myelopathy and leukodystrophy of ALD share several characteristics, including abnormal activation of microglia and early axonal degeneration. Patients presenting with spinal cord disease in adulthood can still develop a leukodystrophy, which illustrates how these disease manifestations should not be regarded as static phenotypes but rather as a disease spectrum. Based on the included studies, we propose the following working model for the pathogenesis of ALD (Fig. 1).

In the spinal cord, increased VLCFA-levels cause myelin destabilization [9]. VLCFA are toxic to oligodendrocytes [8], impairing their remyelination potential although the myelin sheath can initially still be maintained. Over the course of years, loss of myelin leads to expression of axonal stress markers and degeneration. This leads to a vicious cycle of disintegration of the axon-myelin unit and Wallerian degeneration and represents the core pathology of ALD.

In the leukodystrophy, the same process of axonal degeneration is followed by myelin destabilization and fragmentation. The fragments are engulfed by microglia, migrated peripheral macrophages and astrocytes. These in turn produce pro-inflammatory cytokines, including TNF-alpha [48]. Inflammation results in T-lymphocyte and peripheral phagocyte recruitment and oligodendrocyte dysfunction and death. As oligodendrocytes cannot adequately maintain myelin or engage in remyelination, further disintegration of the axon-myelin unit will occur. This process is accelerated by the toxic effects of VCLFA on microglia that cannot sufficiently nurture axons or degrade VLCFA and myelin fragments [50]. Whether a patient develops the leukodystrophy depends on VLCFA levels, environmental, epigenetic and genetic factors and on the integrity of the BBB [57, 67, 82, 83]. A natural predisposition towards the production of anti-inflammatory cytokines and upregulation of other proteins that are able to metabolize VLCFA could have a protective effect [56].

Although this working model cannot describe ALD pathology in all its complexity, the included studies and the proposed theory emphasize several aspects. Most importantly, the pathology of the leukodystrophy and the myelopathy of ALD are different, but in many ways also similar. In both cases, axons are impacted early on in the disease. In the spinal cord, the axonopathy leads to Wallerian degeneration, whereas in cerebral ALD it results in myelin loss and subsequent inflammation. Secondly, the activation pattern of microglia is abnormal with compelling suggestions for their primary involvement in both disease manifestations. Both axonal degeneration and microglia dysfunction should therefore be prioritized in future research. A pending question is how exactly axons are affected by ABCD1 dysfunction, as myelin loss is concurrent to and sometimes preceded by axonal destruction [52]. Although microglia probably have a protective role in the development of cerebral ALD, it is unlikely that they are the only factor influencing axonal degeneration. Significantly fewer studies have been performed on the myelopathy of ALD than on its cerebral counterpart. Given the pathological similarities and overlapping biochemical defect in ALD leukodystrophy and myelopathy, in-depth analysis of spinal cord samples may represent a valuable means to further uncover ALD pathogenesis. Although this review has focused on the CNS, the same holds true for the fascinating tissue specific vulnerability of the adrenal glands.

Although neurons and microglia have in the past received the greatest attention, other glia should not be overlooked in ALD research. For example, relatively few reports have focused on the pathological features of astrocytes in ALD lesions. Astrocytes play a key role in maintaining the BBB and nurture and provide structural support to axons. They respond to tissue injury by orchestrating a multicellular response, thereby also limiting inflammation [84]. In ALD, they express HSPs and inflammatory cytokines. Moreover, astrocytes are among the cells with the highest ABCD1 expression in the CNS. It is therefore plausible that they are also involved in ALD pathogenesis. Induced pluripotent stem cell-derived astrocytes from patients with ALD contain higher (saturated) VLCFA-levels and produced more proinflammatory cytokines than controls [18, 85]. Astrocytes have been associated with a large number of other leukodystrophies. Currently, in the 2017 pathological classification proposed by van der Knaap and Bugiani, cerebral ALD is classified as a demyelinating disease [86]. We argue that although white matter loss is a hallmark of ALD, its pathological origin probably lies elsewhere.

The clinical course of ALD is well-defined, but its pathophysiology remains poorly understood. Few explanatory “grand unifying theories” have been proposed. With the recent advance of modern techniques such as multi-omics and organoids, revisiting the seminal works on pathology could help fill knowledge gaps and refocus research priorities.

### Electronic supplementary material

Below is the link to the electronic supplementary material.

Supplementary Material 1

Supplementary Material 2

Supplementary Material 3

Supplementary Material 4

## Data Availability

Not applicable.

## Abbreviations

ALD: X-linked adrenoleukodystrophy
ALDRP: adrenoleukodystrophy-related protein
AMN: adrenomyeloneuropathy
BBB: blood-brain barrier
CNS: central nervous system
HSCT: hematopoietic stem cell transplantation
IFN-gamma: interferon-gamma
IL-1: interleukin-1
LPC: lysophosphatidylcholine
MHC: major histocompatibility complex
MRI: magnetic resonance imaging
MS: multiple sclerosis
NAWM: normal-appearing white matter
PMP70: 70 kDa peroxisomal membrane protein
PNS: peripheral nervous system
ROS: radical oxygen species
TNF-alpha: tumor necrosis factor-alpha
VLCFA: very-long chain fatty acids
