# Supplementary material for: The pathology of X-linked adrenoleukodystrophy: tissue specific changes as a clue to pathophysiology
Source: Orphanet J Rare Dis. 2024 Mar 28;19:138. doi: 10.1186/s13023-024-03105-0 (PMC10976706; doi:10.1186/s13023-024-03105-0)
Supplement: Supplementary file 1 — Supplementary Material 1 [file 13023_2024_3105_MOESM1_ESM.docx]

Table 1: Characteristics of spinal cord lesions in adrenoleukodystrophy

| Axons and myelin | Axonal degeneration equal to or greater than myelin loss. |
| --- | --- |
| Oligodendrocytes | Reduced in number. |
| Astrocytes | Isomorphic astrogliosis without reactive astrocytes. |
| Macrophages | PAS-positive macrophages abundant in perivascular spaces. |
| Microglia | Numerous activated microglia. |
| T-lymphocytes | Sparsely present as perivascular infiltrates. |

Summary of the results presented by Powers et al. (2000). The following commercial antibodies were used for the stainings: neurofilament (NF), proteolipid protein (PLP), amyloid precursor protein (APP), glial fibrillary acidic protein (GFAP) and the microglia-macrophage markers HAM-56, CD68, 25FD, Ricinus, Lysozyme and non-specific esterase.
