# Supplementary material for: The pathology of X-linked adrenoleukodystrophy: tissue specific changes as a clue to pathophysiology
Source: Orphanet J Rare Dis. 2024 Mar 28;19:138. doi: 10.1186/s13023-024-03105-0 (PMC10976706; doi:10.1186/s13023-024-03105-0)
Supplement: Supplementary file 2 — Supplementary Material 2 [file 13023_2024_3105_MOESM2_ESM.docx]

|  | Normal-appearing white matter | Prelesional zone | Actively demyelinating zone | Gliotic core |
| --- | --- | --- | --- | --- |
| Myelin | Normal. | No relevant myelin loss, myelin vacuolization. | Significant myelin breakdown. Few residual myelinated axons. | (Almost entirely) absent. Remnants vacuolized. |
| Axons | Normal. | Normal morphology, but immunohistochemical signs of damage^1^. | Substantial loss of axons. | (Almost entirely) absent. |
| Astrocytes | Normal. | Occasional reactive cells . | Numerous reactive cells, pattern of anisomorphic gliosis. | Scattered reactive astrocytes, pattern of isomorphic gliosis. |
| Oligodendrocytes | Normal. | Mild oligodendrocyte loss, cells have condensed nuclei. | Significant oligodendrocyte loss. | (Almost) entirely absent, altered morphology with few or no processes. |
| Microglia | Higher numbers with initial reactive morphology. Expression of homeostatic markers. | Almost entirely absent. | Significant microgliosis. | Return to near-normal activation state, with expression of homeostatic markers*.* |
| Macrophages | Absent. | Sparse macrophages, mainly in perivascular spaces. | Drastic increase in lipid-laden foamy macrophages containing degraded myelin fragments. | Persisting slight increase in numbers, as compared to NAWM. |
| Lymphocytes | Absent. | Absent. | Prominent perivascular cuffings of T-lymphocytes. Sparse B-lymphocytes and plasma cells. | Rare T-lymphocytes. |

Table 2: Characteristics of cerebral adrenoleukodystrophy lesions

^1^ Axonal damage was assessed by staining for amyloid precursor protein (APP) and Bielschowsky silver impregnation. The presented results were extracted from studies by Schaumburg et al. (1974 and 1975), Powers et al. (2000), Ito et al. (2001), Eichler et al. (2008) and Bergner et al. (2019 and 2021).
