# Supplementary material for: The pathology of X-linked adrenoleukodystrophy: tissue specific changes as a clue to pathophysiology
Source: Orphanet J Rare Dis. 2024 Mar 28;19:138. doi: 10.1186/s13023-024-03105-0 (PMC10976706; doi:10.1186/s13023-024-03105-0)
Supplement: Supplementary file 3 — Supplementary Material 3 [file 13023_2024_3105_MOESM3_ESM.docx]

**Reviewer #1:**

*Title: „The pathology of X-linked adrenoleukodystrophy: tissue specific changes as a clue to pathophysiology". Main message: While substantial progress has been made in understanding the pathophysiology of X-ALD, the precise sequence of events leading to myelopathy and neuroinflammation remains enigmatic. In this comprehensive review, the authors aim to integrate histochemical observations starting from the 1970s with contemporary insights derived from advanced technologies. Thus, the interesting aspect of the current review by Yska and colleagues is their aim to present an integrative perspective, which will be of clear value to the field.*

- Thank you for your comments. Indeed, we hope that the integrative approach of the manuscript will help elucidate the pathology of X-ALD.

*Specific issues:*

*1) Previously no good markers were available to differentiate between macrophages and microglia within the actively demyelinating zone. Accordingly it would be more appropriate to speak in this context of older studies of lipid-laden phagocytes (eg. p.10, lines 1-5; p. 11, lines 23-26; p. 11, line 54).*

- Although we agree with the reviewer that differentiation between macrophages and microglia must have been difficult at the time due to the absence of histochemical stainings, the publication by Schaumburg et al. (1975) states that lipid-laden *macrophages* are present. Although this may have been incorrect, it could also be argued that the researchers differentiated between cell types based on morphology. If the reviewer agrees, in order to remain as objective as possible, we would prefer to leave the majority of the terminology as it is. We have elaborated on the distinction of different phagocytes on page 10 of the manuscript.

*2) It would be informative if the authors could use publically available novel transcriptomics/proteomics data to complement previously obtained expression data for ABCD1, also in respect to specific cell types such as astrocytes or oligodendrocytes and to different brain regions.*

- Thank you for this suggestion. We agree that a better understanding of the cellular distribution of the ABCD1 protein would aid in the understanding of the pathology of ALD. We however have not been able to find any transcriptomics or proteomics data on the ABCD1 distribution, including at the single cell level. Hoftberger et al. in their publication from 2007 did include some information on mRNA data. This has been added to the manuscript on page 7.

*3) The authors should correct the discrepancy between text (p. 8, line 39) and Table 1 concerning the presence of macrophages within degenerative spinal cord lesions of AMN patients.*

- We have fixed this discrepancy in Table 1 and on page 8.

*4) In the conclusion section, p. 16 lines 31-39 and within Figure 1, the authors should clarify that due to astrocyte/microglial activation, pro-inflammatory cytokines/chemokines are secreted and result in the infiltration of peripheral immune cells (monocytes and T cells), with monocytes differentiating in the parenchyma to macrophages (in other words from the text and cartoon one could get the impression that macrophages derived from the periphery are always present in the brain)*

- Thank you for this suggestion. We have added further information on the origin of the macrophages on page 17 of the manuscript and in the legend of Figure 1.

*5) The authors should add a figure legend to Fig. 1*

- A legend has been added to Figure 1.

*6) In Table 1 it would be informative to add the respective immunohistochemical markers used by Powers et al.*

- Sadly, Powers and colleagues were not very specific in their descriptions on how they identified different cell types. The main markers described in the original manuscript have been added in the legend of Table 1.

*7) In Table 2 references should be added.*

- The appropriate references have been added to the legend of Table 2.

*Minor issues:*

*- The authors should use the new nomenclature for the ABCD1-encoded protein which is ABCD1 and not ALDP*

- ALDP has been replaced by ABCD1 throughout the document.

*- correct the misspelling of "very-long chain" in line 5 and Figure 1 to "very long-chain"*

- This correction has been made accordingly.

**Reviewer #2:**

*The review by Yska and colleagues entitled "The pathology of X-linked adrenoleukodystrophy: tissue specific changes as a clue to pathophysiology" is a easy-to-read summary of the current state of knowledge. An attempt is made to develop a comprehensive pathological concept based on old and new pathological findings. This review represents an important contribution to the understanding of the disease.*

- We thank the reviewer for the compliments and their valuable feedback.

*A few suggestions:*

*1. Page 6 lain 25: "Although ALDRP resembles ALDP structurally, it has a different tissue distribution and does not influence ALD disease phenotype (22, 23)." I think that's too brief and hard to understand to the extent that it is. My suggestion would be, for example: "Although ALDRP resembles ALDP structurally most closely and can compensate for the lack of ALDP by overexpression in vivo and in vitro (Ref: PMID: 10196381 and 15489218, PMC4177892), it has a different tissue and cell type distribution and thus is likely not a major disease modifying factor (22, 23).*

- We understand the reviewer’s comment and have adopted the proposed sentence in the manuscript on page 6. The proposed references have also been included.

*2. Page 8: "Degeneration of the spinal cord is especially prominent in the cervical fasciculus gracilis and the lumbar corticospinal tracts". With regards to the cervical fasciculus gracilis it might be nice to add that this is in very good agreement with the early sensory loss in the lower limbs (possibly not all readers are so well trained in neuroanatomy).*

- Thank you for this suggestion. We have added information on the neurophysiological correlate between these tracts and propriocepsis on page 8.

*3. Page 8 line 54: I assume the authors would like to change the sentence in:…observed, but „activated" astrocytes and ….*

- This sentence has been changed accordingly on page 10.

*4. Page 9 line 58: At the time of Schaumberg there was no discrimination possible between microglia and macrophages. I would suggest that the authors wish to say: and scattered activated microglia and astrocytes.*

- Although we agree with the reviewer, the publication by Schaumburg et al. (1975) states that lipid-laden *macrophages* are present. Although this may have been incorrect, it could also be argued that the researchers differentiated between cell types based on morphology. If the reviewer agrees, in order to remain as objective as possible, we would prefer to use the term “phagocytes” instead of microglia in this sentence. We have elaborated on the distinction of different phagocytes on page 10 of the manuscript.

*5. Page 10, line 41: Hear the authors can possibly could add: This is in very good agreement with the finding the NFL, a marker for axonal damage, appear to be a very early marker in the development of the cerebral form of X-ALD (PMID: 37683329).*

- This sentence and citation have been added at the correct location on page 10.

*6. Page 13 line 5. It might be added that also Hematopoietic stem cell gene therapy with a lentiviral vector in X-linked adrenoleukodystrophy (PMID: 19892975) was successful and that only the monocytes/macrophage cell type but not the lymphocytes show a metabolic defect in X-ALD (PMID: 24363066).*

- Thank you for the suggestion to add information on the lentiviral gene therapy. We agree that this strengthens the hypothesis of a pathological connection between cerebral ALD and phagocytes. These citations have been added on page 13 and 14.

*7. Page 13, line 36: "One argument questioning the central role of microglia in ALD spinal cord pathology is that HSCT does not prevent the development of spinal cord disease (58)." Is this what you like to say? Or .. in contrast to the role in the cerebral inflammatory demyelination in the spinal cord pathology of AMN ……*

- This sentence has been changed accordingly.

*8. Page 14, line 33 at the end ref. PMID: 37759733 would perfectly fit.*

- This reference has been added on page 15.
